# Tuning perovskite recombination by hydrogen interstitial oxidation state

**DOI:** 10.1039/d6ra02305c

**Published:** 2026-04-29

**Authors:** Yong Huang, Rongkun Zhou, Xiaoqing Chen, Wencai Zhou, Hui Yan, Zilong Zheng

**Affiliations:** a State Key Laboratory of Materials Low-Carbon Recycling, Beijing Key Lab of Microstructure and Properties of Advanced Materials, College of Materials Science and Engineering, School of Information Science and Technology Key Laboratory Optoelectronics Technology of Ministry of Education, Beijing University of Technology Beijing 100124 China chenxiaoqing@bjut.edu.cn zilong.zheng@bjut.edu.cn; b Hubei Key Lab of Photoelectric Materials and Devices, School of Materials Science and Engineering, Hubei Normal University Huangshi 435002 Hubei China zwc@hbnu.edu.cn

## Abstract

Nonradiative recombination represents a critical performance limitation in perovskites. Combining time-dependent density functional theory (TD-DFT) with nonadiabatic molecular dynamics (NAMD), we elucidate how the oxidation states of hydrogen interstitial defects (H^0^_i_, H_i_^+^, and H_i_^−^) dictate recombination dynamics in FAPbI_3_. The recombination lifetime depends on the oxidation state, varying over three orders of magnitude, from a short time of 0.1 ns (H^0^_i_) to prolonged times of 44 ns (H_i_^+^) and 72 ns (H_i_^−^). H^0^_i_ introduces a deep-level defect state, enhancing nonadiabatic coupling between band edges from 0.35 meV to 1.56 meV. Chlorine passivation (Cl@H^0^_i_) neutralizes H^0^_i_ defects by eliminating the deep trapping state, stabilizing the lattice and reducing nonadiabatic coupling to 0.30 meV. This passivation enhances the carrier lifetime by 2–3 orders of magnitude, ultimately reaching 87 ns. Our findings establish a map of oxidation-state-dependent recombination for hydrogen interstitials and provide atomistic insights for developing defect passivation strategies for high-performance perovskites.

## Abstract 2

Hydrogen interstitial in perovskites show oxidation-state dependence; H^0^_i_ can introduce a deep state enabling nonradiative decay, while Cl passivates the trap, suppresses nonradiative coupling, and restores the carrier lifetime from 0.1 ns to 87 ns.

## Graphical abstract

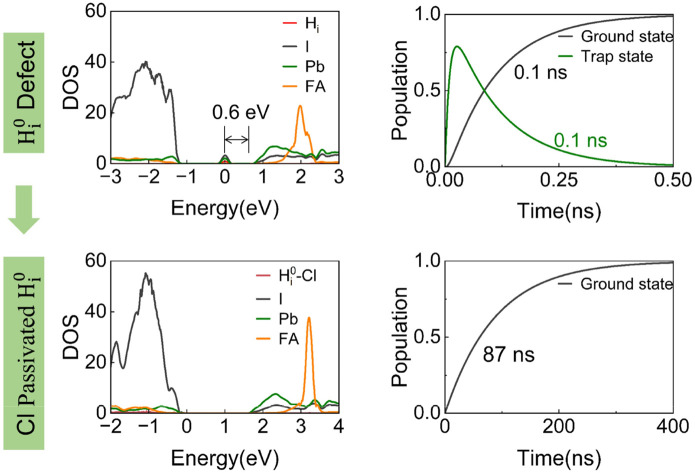

## Introduction

Metal-halide perovskites (MHPs) have emerged as promising light-absorbing materials for photovoltaics, owing to their outstanding optoelectronic properties, including strong optical absorption, long carrier diffusion lengths, and tunable bandgaps.^1,2^ Among various perovskite compositions, formamidinium lead triiodide (FAPbI_3_) has been regarded as a pivotal candidate for high-efficiency perovskite solar cells (PSCs) due to its near-ideal bandgap for single-junction solar cells.^3,4^ Despite remarkable progress, with the power conversion efficiency (PCE) of FAPbI_3_-based devices exceeding 27%,^5^ this still remains below the Shockley–Queisser limit (33%).^6^ A critical pathway toward bridging this efficiency gap lies in suppressing nonradiative recombination losses.

Defect-assisted nonradiative recombination is widely regarded as the dominant loss channel in PSCs. Solution processing and thermal annealing introduce intrinsic point defects, which act as trapping and recombination centers, thereby elevating charge carrier loss and impairing long-term device stability.^7^ While the properties of inorganic lead- and iodine-related defects have been extensively studied,^8,9^ recent evidence highlights the crucial, yet previously underestimated, role of hydrogen-related defects in governing nonradiative recombination dynamics. For instance, hydrogen vacancies resulting from organic cation deprotonation were identified as a key factor affecting the performance of methylammonium lead iodide (MAPbI_3_) under iodine-rich conditions.^10^ Furthermore, associated hydrogen impurities have been shown to dominate nonradiative recombination losses in FAPbI_3_.^11^ Specifically, hydrogen interstitials (H_i_) can exist in multiple oxidation states^12^ (H^0^_i_, H_i_^+^, and H_i_^−^), analogous to iodine vacancies (I_V_) and iodine interstitials (I_i_).^13,14^ These distinct oxidation states are therefore expected to exhibit different electronic structures and nonradiative recombination activities. However, a dynamic understanding of charge-state-dependent H_i_ behavior in FAPbI_3_ is still lacking. In particular, the identity of the dominant recombination-active charge state and the origin of the distinct recombination behaviors of these charge states remain unclear.

Currently, numerous defect passivation strategies have been developed to mitigate these losses, including Lewis acid–base interactions, chemical passivation, ionic compensation, strain engineering, and halogen-assisted defect regulation.^15–20^ In practice, in high-performance devices, mixed-halide compositions are routinely employed, and halogen species such as chlorine (Cl) are often unintentionally incorporated.^21^ While Cl inclusion has been empirically shown to improve device efficiency and stability, the atomistic mechanism by which Cl interacts with hydrogen-related defects to suppress nonradiative recombination remains poorly understood.

In this study, we employ time-dependent density functional theory (TD-DFT) combined with nonadiabatic molecular dynamics (NAMD) to unravel the influence of hydrogen interstitial oxidation states on nonradiative recombination dynamics in FAPbI_3_. We identify the neutral hydrogen interstitial (H^0^_i_) as the dominant recombination-active species governing the nonradiative decay, while the charged states (H_i_^+^ and H_i_^−^) have a minimal or even suppressive effect. By establishing H^0^_i_ as the primary detrimental defect, we further elucidate the passivation mechanism induced by halogen incorporation. Our findings reveal that chlorine passivates H^0^_i_ (Cl@H^0^_i_) by forming a chemical bond with H^0^_i_, which eliminates the deep-level trapping state and weakens electron-phonon coupling, resulting in a significant recovery of the carrier lifetime. This work provides a map of the oxidation-state-dependent recombination mechanisms of hydrogen impurities and delivers a microscopic outlook for designing effective passivation strategies to achieve high-performance PSCs.^22–24^

## Computational methods

We employed the dephasing-induced surface hopping (DISH) method implemented within the *ab initio* time-dependent density functional theory (TD-DFT) framework^25^ in combination with nonadiabatic molecular dynamics (NAMD) simulations to investigate carrier capture and nonradiative recombination processes in pristine FAPbI_3_, H^0^_i_, H_i_^+^, H_i_^−^, and Cl@H^0^_i_ systems. Owing to the much slower motion of atomic nuclei compared to electrons, the nuclear degrees of freedom were treated semi-classically within classical mechanics, while the electronic subsystem was described quantum mechanically. The DISH algorithm enables trajectory branching, simulating surface hopping during dephasing events. Given that the quantum coherence time induced by phonons is much shorter than the electron–hole recombination time, dephasing effects are incorporated into the NAMD simulations.^26^ The dephasing time, similar to the pure decoherence time in light-response theory, can be approximated by second-order cumulants.^27^ This method has been widely applied to the study of photoexcited charge dynamics in perovskites.^28,29^

All simulations were performed using a 2 × 2 × 2 supercell containing 96 atoms, which is sufficient to model hydrogen-related point defects in the bulk phase. The H^0^_i_ configuration was obtained by adding a hydrogen atom to the pristine FAPbI_3_ lattice. The H_i_^+^ and H_i_^−^ systems were generated by respectively adding or removing an electron from the H^0^_i_ structure. The Cl@H^0^_i_ system was obtained by introducing a Cl atom into the H^0^_i_ system. Geometry optimization, *ab initio* molecular dynamics (AIMD), and nonadiabatic (NA) coupling calculations were performed using the first-principles simulation package VASP.^30^ Exchange-correlation effects were described using the Perdew–Burke–Ernzerhof (PBE)^31^ functional, and electron-ion core interactions were treated using the projector augmented wave (PAW) method.^32^ During geometry optimization, a 3 × 3 × 3 Monkhorst–Pack grid was used, with an energy cutoff of 400 eV to ensure total energy convergence. Long-range van der Waals interactions were accounted for using Grimme's DFT-D3 correction.^33^ The NAMD calculations were performed using the Hefei-NAMD program.^34^ Based on the optimized geometries at 0 K, the system was equilibrated at 300 K for 3 ps with the *NVT* ensemble, followed by another 6 ps in the *NVE* ensemble, and the trajectories from the last 3 ps were collected for NA coupling and thermal fluctuation calculations.^35^ We ran 300 ps NAMD simulations for each pair of states to calculate the rate constants for the electron–hole capture process.

## Results and discussion

### Structural modifications and lattice dynamics induced by H_i_

**Fig. 1:**
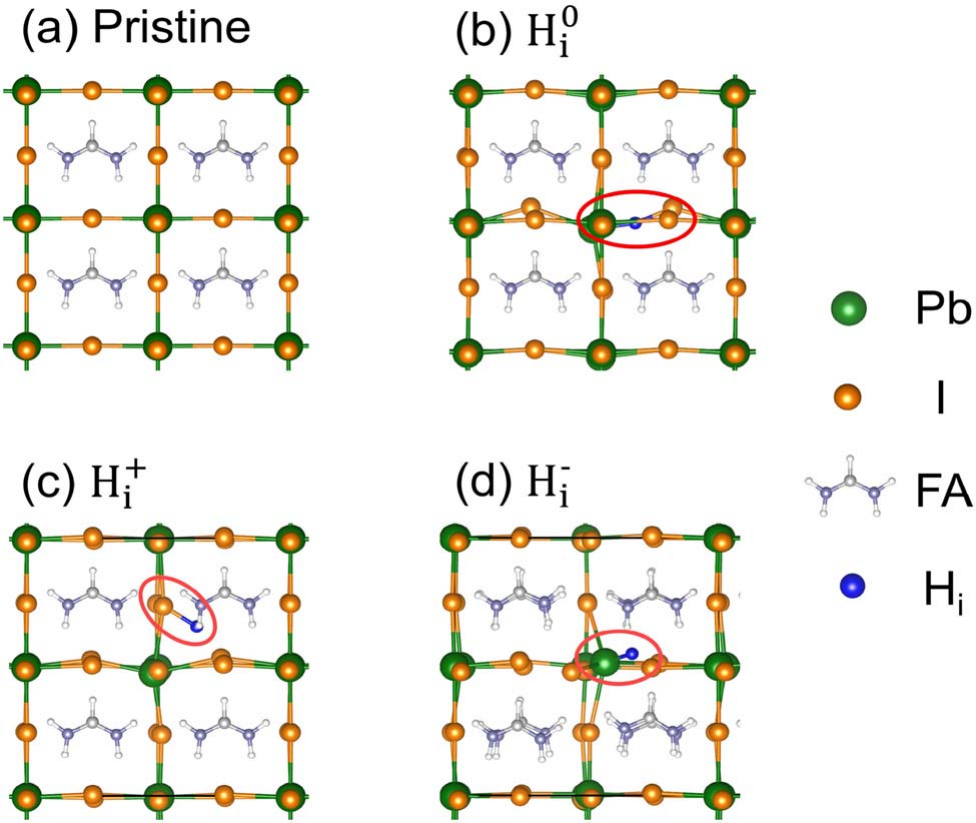
Configurations of (a) pristine FAPbI_3_ and systems with (b) neutral H^0^_i_, (c) positive H_i_^+^, and (d) negative H_i_^−^ defects.

The introduction of H_i_ defects into the FAPbI_3_ lattice leads to oxidation-state-dependent structural distortions. While the pristine inorganic Pb–I lattice exhibits rigidity (see Fig. 1a), each oxidation state of H_i_ adopts a distinct local coordination: neutral H^0^_i_ occupies a bridge site along a Pb–I bond (see Fig. 1b); H_i_^+^ interacts with iodide anions through coulomb attraction (see Fig. 1c); and H_i_^−^ coordinates with Pb^2+^ cations, forming a localized fourfold-coordinated octahedral environment (see Fig. 1d). These configurations influence the dynamic disorder of the lattice.

**Fig. 2:**
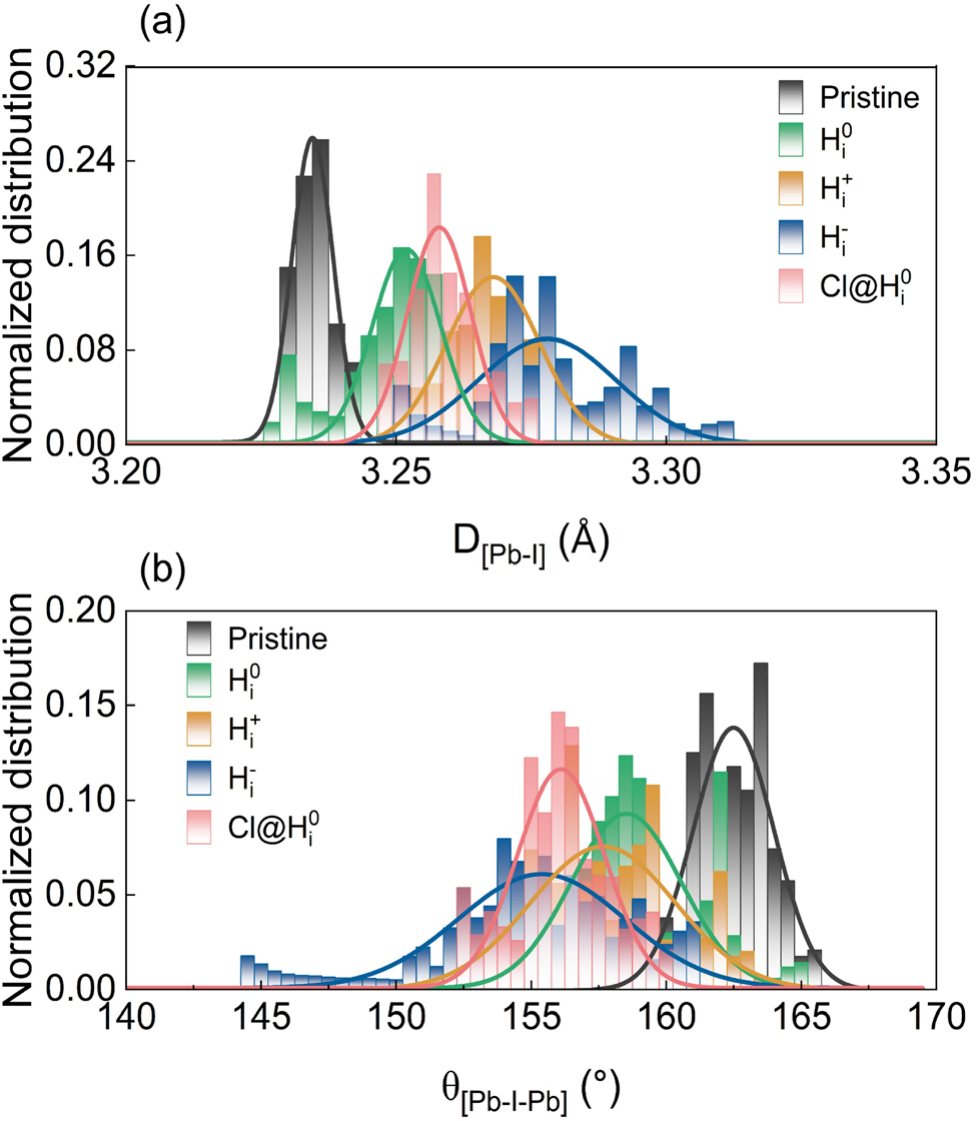
Statistical distributions of (a) the Pb–I bond lengths (*D*_[Pb–I]_) and (b) the Pb–I–Pb bond angles (*θ*_[Pb–I–Pb]_) in pristine FAPbI_3_ and systems containing neutral H^0^_i_, positive H_i_^+^, negative H_i_^−^, and chlorine-passivated H^0^_i_ (Cl@H^0^_i_) defects. The histograms were obtained from molecular dynamics trajectories at 300 K and fitted with Gaussian functions (solid lines) to extract the mean values and standard deviations.

To quantify the effect, we explored the distributions of the Pb–I bond lengths (*D*_[Pb_–_I]_) and Pb–I–Pb bond angles (*θ*_[Pb_–_I_–_Pb]_) derived from molecular dynamics simulations, as shown in Fig. 2, and the mean values and standard deviations are provided in Table S1 of the supplementary information (SI). The mean values reflect the average bond lengths and angles, while the standard deviation quantifies the degree of thermodynamic perturbation in the lattice. All H_i_ defects enhance structural fluctuations compared to the pristine system, as reflected by the standard deviations of the bond lengths (*σ*_bond_), which increase in the order of *σ*_bond_ (pristine) < *σ*_bond_ (H^0^_i_) < *σ*_bond_ (H_i_^+^) < *σ*_bond_ (H_i_^−^). The H_i_^−^ system exhibits the most pronounced distortion, which is attributed to the strong attraction between the hydrogen anion and adjacent Pb^2+^ cation, leading to the rupture of two Pb–I bonds. In contrast, H_i_^+^ and H^0^_i_ result in milder octahedral distortion, making the structures more stable compared to the H_i_^−^ system. The octahedral distortion is related to enhanced charge localization, decreasing the carrier recombination rate.^9^

To quantitatively evaluate lattice stability and thermal vibrations, we obtained the root mean square velocities, as shown in Fig. S1 in the SI. This further confirms that H_i_ incorporation enhances atomic thermal motion, particularly within the inorganic lattice, thereby accelerating decoherence and influencing carrier recombination dynamics, which correlates with the carrier lifetimes.^9^

### Electronic structure modifications and defect-state formation

**Fig. 3:**
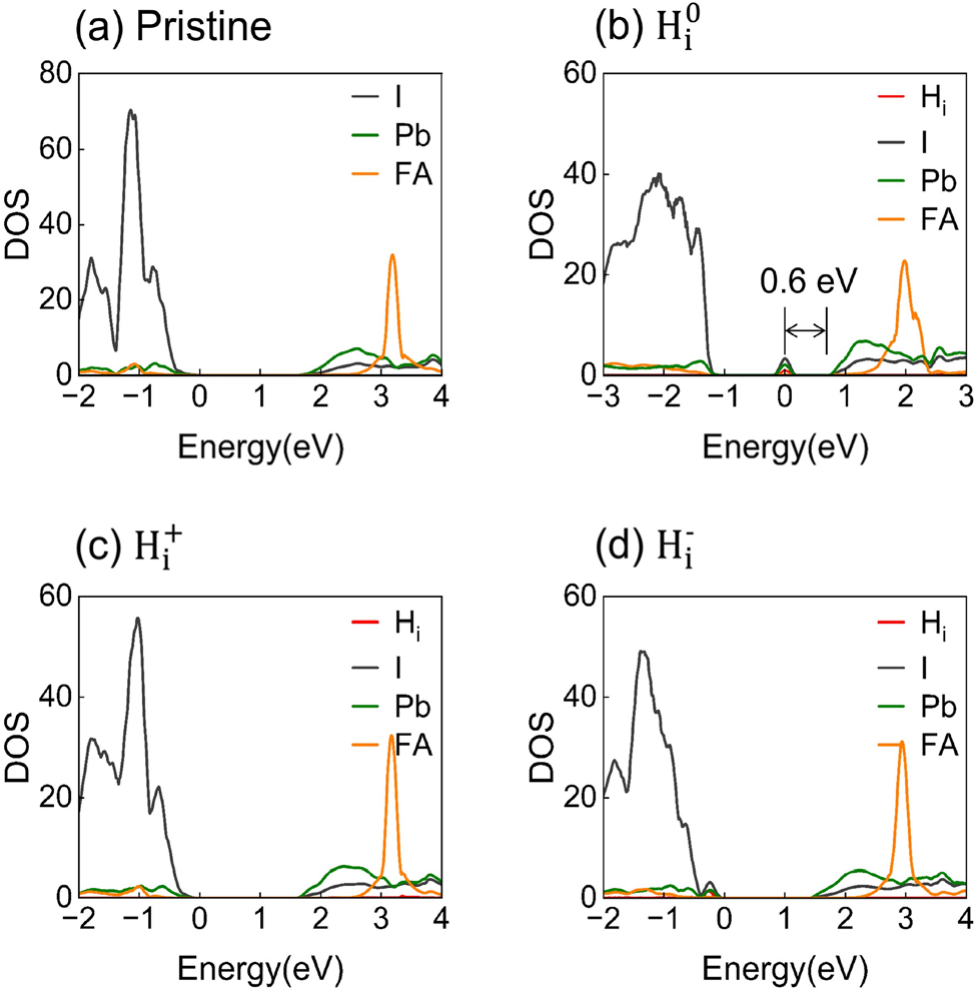
Projected density of states (PDOS) for (a) pristine FAPbI_3_ and systems containing (b) neutral H^0^_i_, (c) positive H_i_^+^, and (d) negative H_i_^−^ defects; only H^0^_i_ introduces a deep-level state within the bandgap of FAPbI_3_.

The electronic properties of FAPbI_3_ are strongly modulated by the oxidation state of interstitial hydrogen. As illustrated by the projected density of states (PDOS) in Fig. 3, the pristine FAPbI_3_ system shows a clean bandgap of 1.7 eV. The introduction of H^0^_i_ produces a deep-level trapping state 0.6 eV below the conduction band minimum (CBM), originating from the Pb–H^0^_i_–I trimer configuration, as shown in Fig. S2 in the SI. This state acts as an efficient nonradiative recombination center. In contrast, H_i_^+^ yields no mid-gap defect state, and H_i_^−^ introduces only a shallow defect state above the valence band maximum (VBM). These results highlight the critical role of the defect charge state in determining the electronic landscape and subsequent recombination pathways.

### Electron–phonon coupling and decoherence dynamics

To characterize the phonon modes coupled to electronic transitions between electronic states, we calculated the autocorrelation function (ACF) of the energy-gap fluctuations and its Fourier transform (FT). Within the framework of optical response theory, the ACF provides access to the pure dephasing time, while the FT spectrum, referred to as the spectral density or influence spectrum, reflects the frequency-dependent electron-phonon coupling strength. Each peak intensity represents the coupling strength at a specific phonon frequency.

**Fig. 4:**
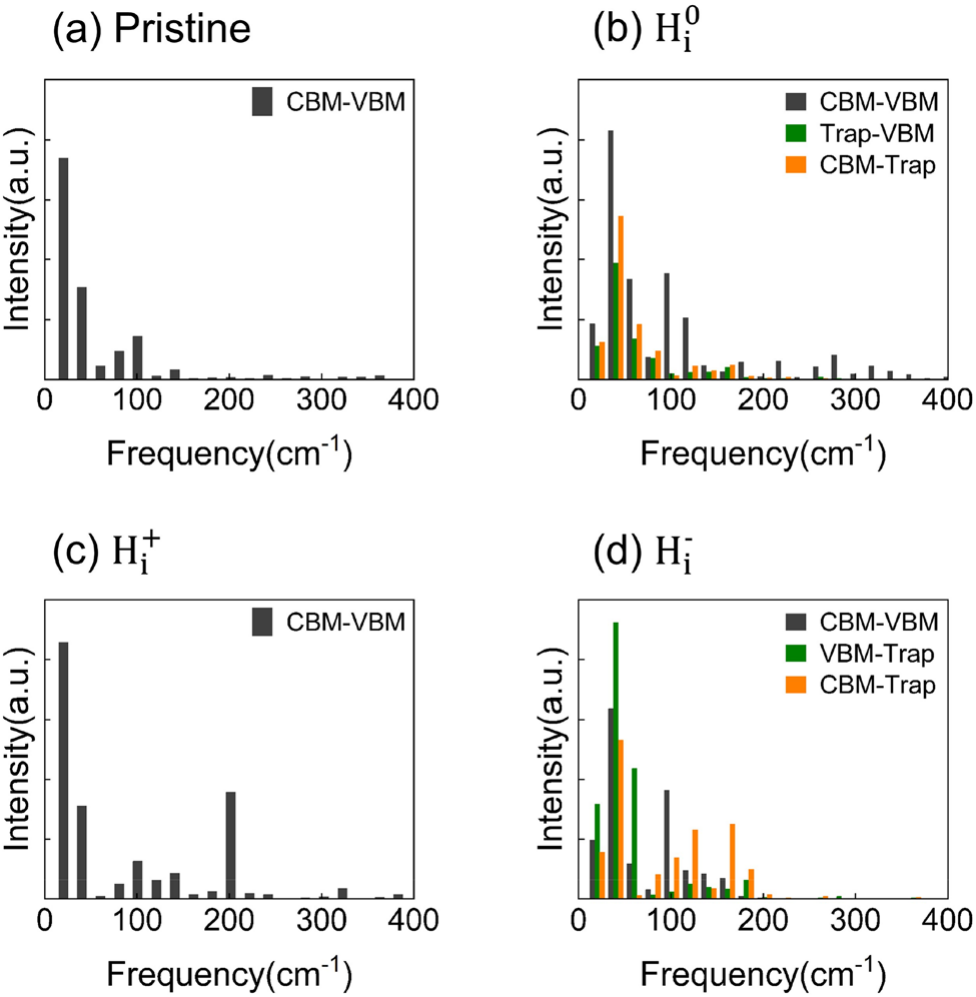
Spectral densities of energy-gap fluctuations for VBM/CBM, VBM/trapping-state, and CBM/trapping-state in (a) pristine FAPbI_3_ and systems with (b) neutral H^0^_i_, (c) positive H_i_^+^, and (d) negative H_i_^−^ defects, derived from Fourier transforms of the autocorrelation functions.

The spectral densities in Fig. 4 are dominated by low-frequency modes arising from the atomic motion of Pb and I atoms, which facilitate charge capture and recombination. Peaks below 100 cm^−1^ are primarily attributed to the stretching and bending vibrations of Pb–I bonds,^36^ which play a crucial role in nonadiabatic (NA) coupling. The NA coupling values are summarized in Table 1 and Table S2 of the SI. The presence of H_i_ enhances these low-frequency (below 100 cm^−1^) vibrational contributions due to increased octahedral distortion. Specifically, the H_i_^−^ system exhibits pronounced structural distortions that intensify low-frequency modes, with a marked enhancement of the peak near 40 cm^−1^. Phonon modes in the 100–200 cm^−1^ range are associated with organic cation motion, and those above 200 cm^−1^ correspond to FA cation torsional modes.^36^ These FA-derived modes can indirectly influence electron-phonon coupling through electrostatic interactions. Hydrogen interstitial defects also break lattice symmetry, creating localized states and high-frequency vibrational features.

**Table 1:** Comparative parameters for nonradiative recombination across pristine, neutral H^0^_i_, positive H_i_^+^, negative H_i_^−^ defect, and Cl-passivated H^0^_i_ (Cl@H^0^_i_) FAPbI_3_ systems, including energy gap, non-adiabatic (NA) coupling, and dephasing time

		Gap (eV)	NA coupling (meV)	Dephasing time (fs)
Pristine	CBM-VBM	1.7	0.39	10.7
H^0^_i_	Trap-VBM	1.2	1.56	2.5
H_i_^+^	CBM-VBM	1.7	0.41	7.2
H_i_^−^	CBM-Trap	1.7	0.35	6.6
Cl@H^0^_i_	CBM-VBM	1.7	0.30	9.4

**Fig. 5:**
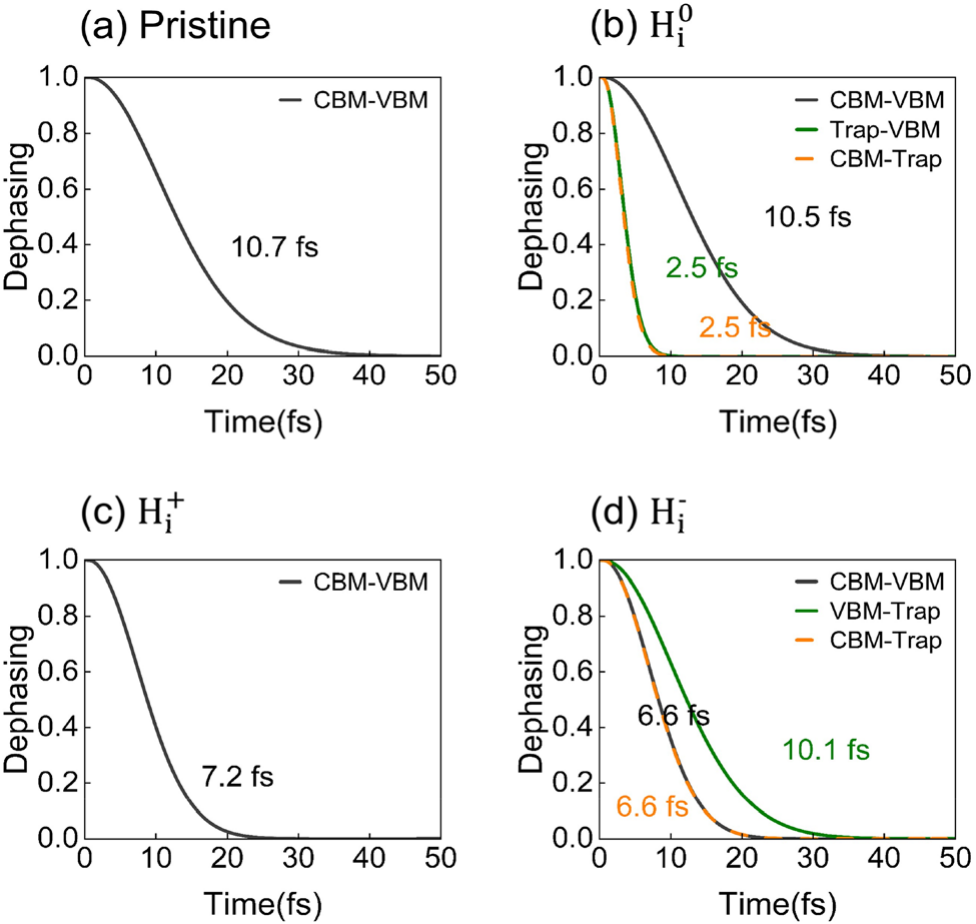
Dephasing functions representing elastic electron-phonon scattering for VBM/CBM, VBM/trapping-state, and CBM/trapping-state in (a) pristine FAPbI_3_ and systems with (b) neutral H^0^_i_, (c) positive H_i_^+^, and (d) negative H_i_^−^ defects.

Fig. 5 presents the pure dephasing functions of the pristine and three H_i_ defect systems, associated with elastic electron–phonon scattering. The pure dephasing time was obtained *via* Gaussian fitting with the expression *f*(*t*) = e^−0.5(*t*/*τ*)2^. For all systems, pure dephasing occurs on an ultrafast timescale of approximately 10 fs, which is significantly shorter than the timescales for charge capture and recombination. As a result, decoherence effects must be explicitly considered in nonadiabatic molecular dynamics (NAMD) simulations. The pure dephasing time for CBM-VBM transitions in pristine FAPbI_3_ is 10.7 fs (see Fig. 5a). Introducing H_i_ defects activates additional vibration modes, thereby shortening the dephasing time to 2.5 fs for neutral H^0^_i_, 7.2 fs for positive H_i_^+^, and 6.6 fs for negative H_i_^−^, as shown in Fig. 5b–d and Table 1. The shorter dephasing times reflect the high mobility and strong vibrational activity of H_i_. It should be noted that this trend is not universal across all H_i_ defects, underscoring the importance of considering inelastic electron–phonon coupling.

The electron–phonon coupling involves both elastic and inelastic scattering processes. Inelastic scattering is governed by nonadiabatic coupling (NAC), 〈*ϕ*_*i*_|∇_*R*_|*ϕ*_*f*_〉, which depends on the overlap between the wavefunctions of the initial (*ϕ*_*i*_) and final (*ϕ*_*f*_) states involved in a transition. The NAC values are 0.39 meV for pristine FAPbI_3_, 1.56 meV for H^0^_i_, 0.41 meV for H_i_^+^, and 0.35 meV for H_i_^−^. Notably, NAC increases by a factor of four upon introducing H^0^_i_. This pronounced enhancement stems from the reduced energy gap induced by the emergence of deep-level trapping states, a phenomenon that strongly enhances wavefunction overlap.

### Carrier recombination governed by oxidation state

**Fig. 6:**
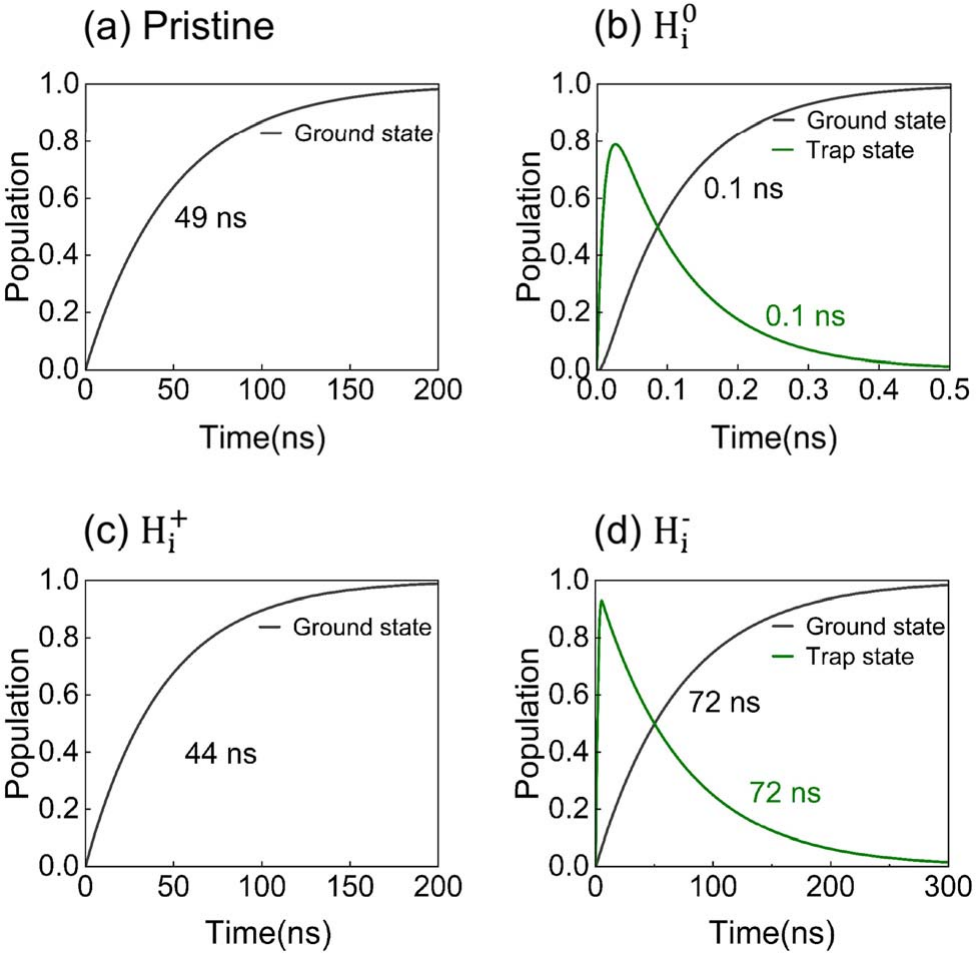
Carrier lifetime dictated by the hydrogen interstitial (H_i_) oxidation state. The time-dependent populations of electronic states reveal that the nonradiative recombination lifetimes vary by three orders of magnitude, from 0.1 ns to 72 ns, in (a) pristine FAPbI_3_ and systems containing (b) H^0^_i_, (c) H_i_^+^, and (d) H_i_^−^ defects.

To determine the transition rates between electronic states, 300 ps NAMD simulations were performed. These rates were incorporated into kinetic equations, with analytical solutions describing state populations (details are provided in the SI). The outcomes of state-to-state NAMD simulations are shown in Fig. S3 and S4 of the SI, while Fig. 6 presents rates derived from exponential fitting of simulation data.

The results reveal the strong dependence of charge capture and recombination dynamics on the oxidation state of hydrogen interstitials. Recombination lifetimes span nearly three orders of magnitude, ranging from 0.1 to 72 ns (Fig. 6). In pristine FAPbI_3_, the electron–hole recombination time is 49 ns, consistent with previous reports.^37^ The H^0^_i_ system introduces a deep-level trapping state that activates additional relaxation pathways, thereby boosting the electron–hole recombination rate by over two orders of magnitude. In contrast, H_i_^+^ does not appreciably alter the bandgap and yields a recombination lifetime of 44 ns, comparable to the value of 49 ns in the pristine system. Interestingly, H_i_^−^ extends the recombination lifetime to 72 ns. This behavior shows that the existence of a defect state alone does not necessarily enhance nonradiative recombination. In the H_i_^−^ system, the defect state is shallow and located near the VBM, and therefore, it cannot efficiently mediate band-to-band recombination. In addition, nonadiabatic coupling in the H_i_^−^ system is weaker than in the pristine FAPbI_3_ system, while the stronger local lattice distortion and charge localization further reduce the wavefunction overlap between the initial and final states. These factors collectively suppress nonradiative recombination and lead to the prolonged carrier lifetime.

### Halogen passivation of the H^0^_i_ defect

To evaluate the thermodynamic stability of H_i_ in different charge states, we calculated the formation energies of H^0^_i_, H_i_^+^, and H_i_^−^ as a function of the Fermi level (Fig. S5 in the SI). The results show that H^0^_i_ possesses a finite stability window within the band gap, indicating that it is thermodynamically accessible in FAPbI_3_. Together with our nonadiabatic dynamics results identifying H^0^_i_ as the most recombination-active defect state, this thermodynamic analysis justifies our focus on the passivation of H^0^_i_-induced recombination. As chlorine is inherently present in mixed-halide perovskite compositions, as confirmed by many experimental studies,^20^ we focus on elucidating the passivation mechanism of H^0^_i_ using Cl (Cl@H^0^_i_).

**Fig. 7:**
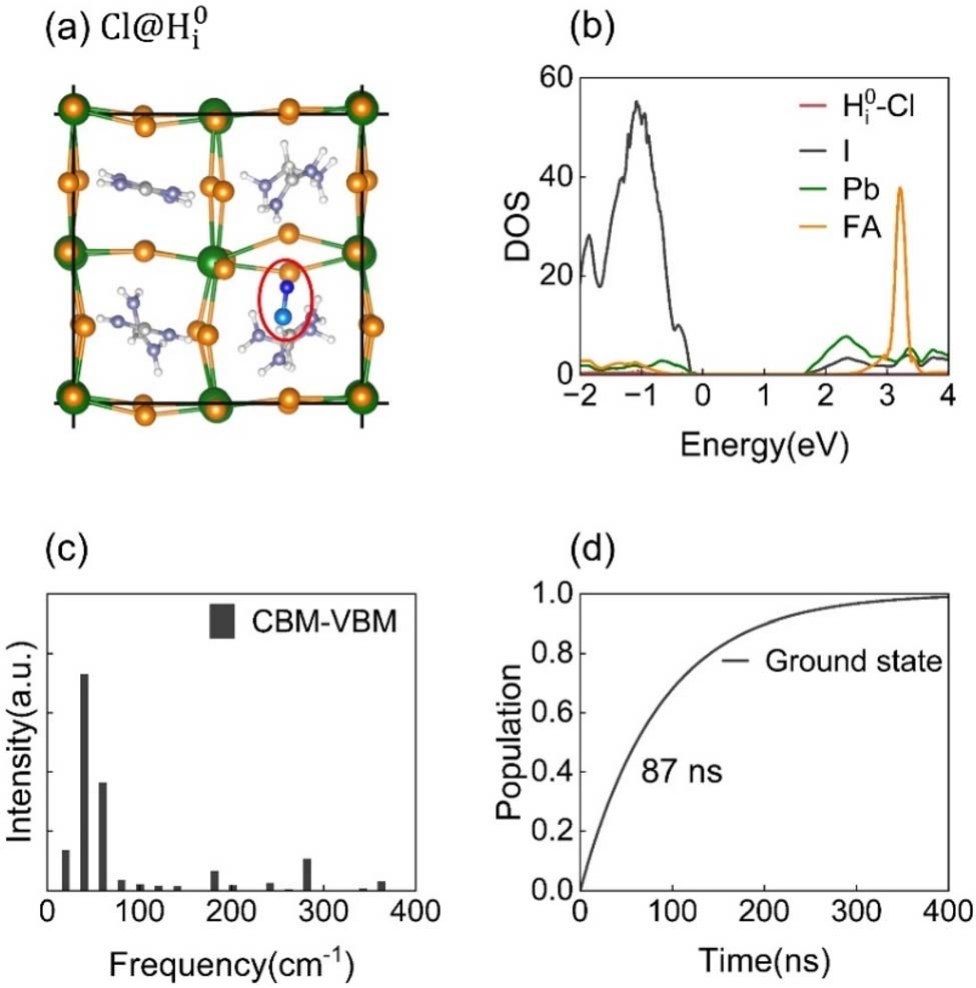
A chlorine-passivated H^0^_i_ defect (Cl@H^0^_i_) in FAPbI_3_. (a) The atomic structure of the Cl@H^0^_i_ complex. (b) Projected density of states (DOS) showing the elimination of the deep-level trapping state. (c) Spectral density of CBM-VBM fluctuations derived from the Fourier transform of the energy-gap autocorrelation function. (d) The time evolution of state populations demonstrating the suppressed nonradiative recombination and prolonged carrier lifetime.

Cl interstitials preferentially bind with H^0^_i_ (Fig. 7a), forming stable Cl–H ionic bonds with a binding energy of 3.1 eV. The high electronegativity of Cl draws the hydrogen interstitial away from the Pb–I octahedral framework, leading to more localized structural distortion. This is reflected in a slight decrease of the Pb–I–Pb bond angle from 159° to 156°, as shown in Fig. 2 and Table S1, which suppresses dynamic lattice fluctuations. Root-mean-square velocity analysis (Fig. S1 in the SI) further confirms that Cl incorporation significantly reduces the thermal motion of Pb, I, and H_i_, thereby stabilizing the perovskite lattice and diminishing time-dependent potential variations linked to strong electron–phonon coupling.

Projected DOS analysis reveals that Cl passivation eliminates the deep-level trapping state induced by H^0^_i_ and shifts its energy toward the valence band, restoring a clean bandgap, as shown in Fig. 7b. The spectral density of VBM-CBM transitions tends to be dominated by low-frequency modes, arising from FA cation torsional vibrations (200–400 cm^−1^). Upon Cl incorporation, the stabilization of the Pb–I framework redistributes the spectral weight toward higher-frequency vibrational modes, as shown in Fig. 7c.

As shown in Fig. 7d, nonradiative recombination in the Cl@H^0^_i_ system is suppressed by 2–3 orders of magnitude compared to the unpassivated H^0^_i_ system, extending the carrier lifetime to 87 ns. This significant improvement stems from a combined reduction in nonadiabatic coupling and prolonged pure dephasing time. Specifically, the NA coupling decreases from 1.56 meV to 0.30 meV, while the dephasing time increases from 2.5 fs to 9.4 fs, as shown in Table 1.

## Conclusions

In summary, we elucidate the critical role of hydrogen interstitial oxidation states (H^0^_i_, H_i_^+^, and H_i_^−^) in governing nonradiative recombination dynamics in FAPbI_3_ perovskites. By combining time-dependent density functional theory with nonadiabatic molecular dynamics simulations, we clarify that the neutral H^0^_i_ defect acts as the dominant recombination center due to its introduction of a deep-level trapping state that significantly enhances nonadiabatic coupling. In contrast, the charged H_i_^+^ and H_i_^−^ defects exhibit minimal or even suppressive effects on recombination. Furthermore, we reveal the effective passivation mechanism of chlorine (Cl), which binds with H^0^_i_ to form a stable complex, eliminating the detrimental deep-level defect state, stabilizing the lattice, and weakening electron–phonon coupling. This passivation strategy successfully restores the carrier lifetime by 2–3 orders of magnitude. These findings provide atomistic insights into the oxidation-state-dependent recombination mechanism and deliver a practical route for designing high-performance perovskite optoelectronic devices through targeted defect control and passivation.

## Author contributions

The project was conceived by XC, WZ and ZZ. XC, WZ, HY and ZZ supervised the project. YH and RZ carried out the theoretical calculations and data analysis. The manuscript was written by YH and RZ with input from all authors. All authors discussed the results and approved the final manuscript.

## Conflicts of interest

There are no conflicts to declare.

## Supplementary Material

RA-016-D6RA02305C-s001

## Data Availability

The data supporting this article have been included as part of the supplementary information (SI). Supplementary information: structural statistics, AIMD analyses, defect charge-density plots, nonradiative recombination parameters, coupled kinetic equations, and carrier population dynamics. See DOI: https://doi.org/10.1039/d6ra02305c.
