# Supplementary material for: Tuning perovskite recombination by hydrogen interstitial oxidation state
Source: RSC Adv. 2026 Apr 29;16(25):22481–7. doi: 10.1039/d6ra02305c (PMC13127114; doi:10.1039/d6ra02305c)
Supplement: RA-016-D6RA02305C-s001 [file RA-016-D6RA02305C-s001.pdf]

Supplementary Information (SI) for RSC Advances.
This journal is © The Royal Society of Chemistry 2026

Supporting Information

Tuning Perovskite Recombination by Hydrogen Interstitial Oxidation State

Yong Huang[1, #], Rongkun Zhou[1, #], Xiaoqing Chen[1, *],
Wencai Zhou[2, *], Hui Yan[1], and Zilong Zheng[1, *].

[1] State Key Laboratory of Materials Low-Carbon Recycling, Beijing Key Lab of Microstructure and Properties of Advanced Materials, College of Materials Science and Engineering, School of Information Science and Technology Key Laboratory Optoelectronics Technology of Ministry of Education, Beijing University of Technology, Beijing 100124, China.

[2] Hubei Key Lab of Photoelectric Materials and Devices, School of Materials Science and Engineering, Hubei Normal University, Huangshi 435002, Hubei, China.

# Yong Huang and Rongkun Zhou contributed equally to this paper.

*Correspondence Author (E-mail: zilong.zheng@bjut.edu.cn; chenxiaoqing@bjut.edu.cn; zwc@hbnu.edu.cn)

Table S1. The mean values (μ) and standard deviations (σ) of the Pb-I bond length ($D_{[Pb-I]}$) and Pb-I-Pb bond angle ($\theta_{[Pb-I-Pb]}$) for pristine $FAPbI_3$, H_i^0, H_i^+, H_i^-, and Cl@H_i^0 systems. The statistical distributions were obtained from AIMD trajectories at 300 K, and the corresponding μ and σ values were extracted by Gaussian fitting of the normalized distributions

		Pristine	H_i^0	H_i^+	H_i^-	Cl@H_i^0
$D_{[Pb-I]}$	μ	3.23	3.25	3.27	3.28	3.26
	σ	0.004	0.009	0.010	0.013	0.007
$\theta_{[Pb-I-Pb]}$	μ	162.5	158.5	157.6	155.4	156.1
	σ	1.50	1.93	2.05	3.08	1.62

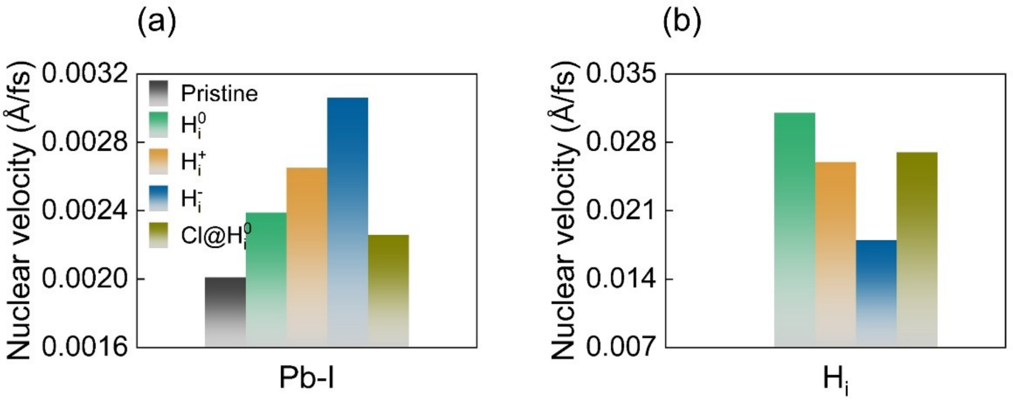

Figure S1. Time-averaged nuclear velocities of Pb and I framework atoms and H atoms in pristine $FAPbI_3$, H_i^0, H_i^+, H_i^-, and Cl@H_i^0 systems.

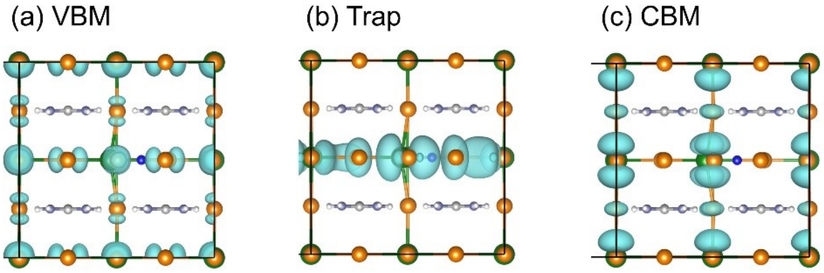

Figure S2. Partial charge densities of the VBM, CBM, and H_i^0-induced defect states.

Table S2. Comparative parameters for nonradiative recombination across pristine $FAPbI_3$, neutral H_i^0 and negative H_i^- defective systems, including energy gaps, non-adiabatic (NA) couplings, and dephasing times.

		Gap(eV)	NA coupling(meV)	Dephasing time (fs)
H_i^0	CBM-VBM	1.8	0.43	10.5
	CBM-Trap	0.6	2.97	2.5
H_i^-	CBM-VBM	1.9	0.30	6.6
	VBM-Trap	0.2	9.36	10.1

The Coupled Kinetics Equations

Here, we consider the following processes that characterize the charge carrier relaxation pathways in pristine $FAPbI_3$, H_i^0, H_i^+, H_i^- and Cl@H_i^0 systems. The schematics of related charge processes are depicted in Figure S3. Here, the transition rate between the conduction band minimum (CBM) and valence band maximum (VBM), CBM and electron trap, electron trap and VBM, VBM and hole trap is denoted by $k_{(CBM\text{-}VBM)}$, $k_{(CBM\text{-}Trap)}$, $k_{(Trap\text{-}VBM)}$, $k_{(VBM\text{-}Trap)}$, respectively.

(a) Pristine $FAPbI_3$, H_i^+, Cl@H_i^0: electron-hole recombination across CBM and VBM. The time-dependent populations of excited state (CBM) and ground state (VBM) are described by equations 1-2 and their solutions are given by equations 3-4. Figure S4a-c gives the state population growing and rate constant.

Coupled kinetic equations:

$$\frac{d[ES]}{dt} = - k_{(CBM - VBM)}[ES] \tag{S1}$$

$$\frac{d[GS]}{dt} = k_{(CBM - VBM)}[GS] \tag{S2}$$

the solutions for this set of equations are:

$$[ES] = e^{- k_{(CBM - VBM)} * t} \tag{S3}$$

$$[GS] = 1 - e^{- k_{(CBM - VBM)} * t} \tag{S4}$$

(b) H_i^0: electron-hole recombination mediated by trap states. The electron-trap mediated

electron hole recombination is described by equations 5-7 and the corresponding solutions are shown in equations 8-10. Figure S5 gives the state population growing and rate constant. Figure S4d gives the state population growing and rate constant.

Coupled kinetic equations:

$$\frac{d[ES]}{dt} = -(k_{(CBM-VBM)} + k_{(CBM-Trap)})[ES] \qquad \text{(S5)}$$

$$\frac{d[Trap]}{dt} = k_{(CBM-Trap)}[ES] - k_{(Trap-VBM)})[Trap] \qquad \text{(S6)}$$

$$\frac{d[GS]}{dt} = k_{(CBM-VBM)}[ES] + k_{(Trap-VBM)})[Trap] \qquad \text{(S7)}$$

the solutions for this set of equations are:

$$[ES] = e^{-(k_{(CBM-VBM)} + k_{(CBM-Trap)}) * t} \qquad \text{(S8)}$$

$$[Trap] = \frac{k_{(CBM-Trap)}}{(k_{(CBM-VBM)} + k_{(CBM-Trap)} - k_{(Trap-VBM)})} e^{-k_{(Trap-VBM)} * t} - e^{-(k_{(CBM-VBM)} + k_{(CBM-T\ldots}}$$ [illegible]

(S9)

$$[GS] = 1 - \frac{1}{(k_{(CBM-VBM)} + k_{(CBM-Trap)} - k_{(Trap-VBM)})}\{k_{(CBM-Trap)} * e^{-k_{(Trap-VBM)} * t} - (k_{(CBM\ldots}$$ [illegible]

(S10)

(c) H_i^-: electron-hole recombination mediated by trap states. The hole-trap assisted charge recombination is described by equations 11-13 and whose solutions are presented in equations 14-16. Figure S4e gives the state population growing and rate constant.

Coupled kinetic equations:

$$\frac{d[ES]}{dt} =- (k_{(CBM-VBM)} + k_{(VBM-Trap)})[ES] \qquad \text{(S11)}$$

$$\frac{d[Trap]}{dt} = k_{(VBM-Trap)}[ES] - k_{(CBM-Trap)})[Trap] \qquad \text{(S12)}$$

$$\frac{d[GS]}{dt} = k_{(CBM-VBM)}[ES] + k_{(CBM-Trap)})[Trap] \qquad \text{(S13)}$$

the solutions for this set of equations are:

$$[ES] = e^{-(k_{(CBM-VBM)} + k_{(VBM-Trap)}) * t} \qquad \text{(S14)}$$

$$[Trap] = \frac{k_{(VBM-Trap)}}{(k_{(CBM-VBM)} - k_{(CBM-Trap)} + k_{(VBM-Trap)})} e^{-k_{(CBM-Trap)} * t} - e^{-(k_{(CBM-VBM)} + k_{(VBM-T}}$$ [illegible]

(S15)

$$[GS] = 1 - \frac{1}{(k_{(CBM-VBM)} - k_{(CBM-Trap)} + k_{(VBM-Trap)})} \{ k_{(VBM-Trap)} * e^{-k_{(CBM-Trap)} * t} - (k_{(CBM}$$ [illegible]

(S16)

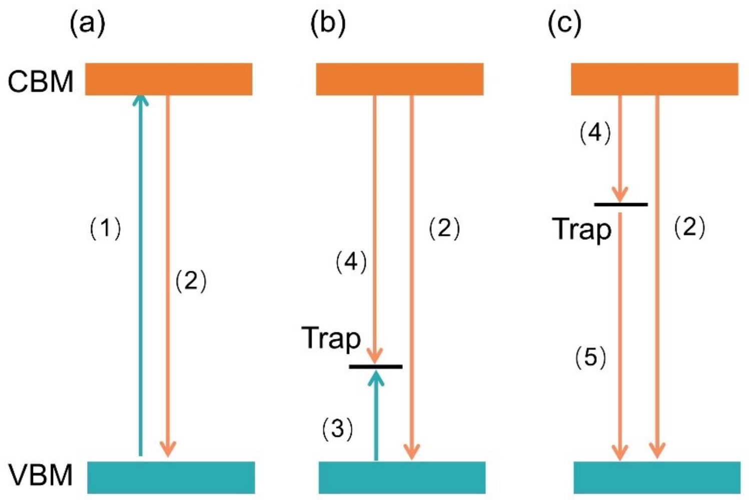

Figure S3. Schematic illustration of charge capture and nonradiative electron-hole recombination processes. Panel (a) depicts the direct band-to-band nonradiative recombination pathway without defect-state involvement, which is applicable to

pristine $FAPbI_3$, H_i^+, and halogen@H_i^0 systems. Panel (b) illustrates the trap-assisted recombination mechanism dominated by hole capture in the H_i^- system, while panel (c) shows the trap-assisted recombination process dominated by electron capture in the H_i^0 system. The numbered processes are defined as follows: (1) photoexcitation generating electrons and holes across the bandgap; (2) nonradiative band-to-band electron–hole recombination between the CBM and VBM; (3) hole capture from the VBM to the trap state; (4) electron capture from the CBM to the trap state; and (5) nonradiative recombination between the trapped electron and holes in the VBM.

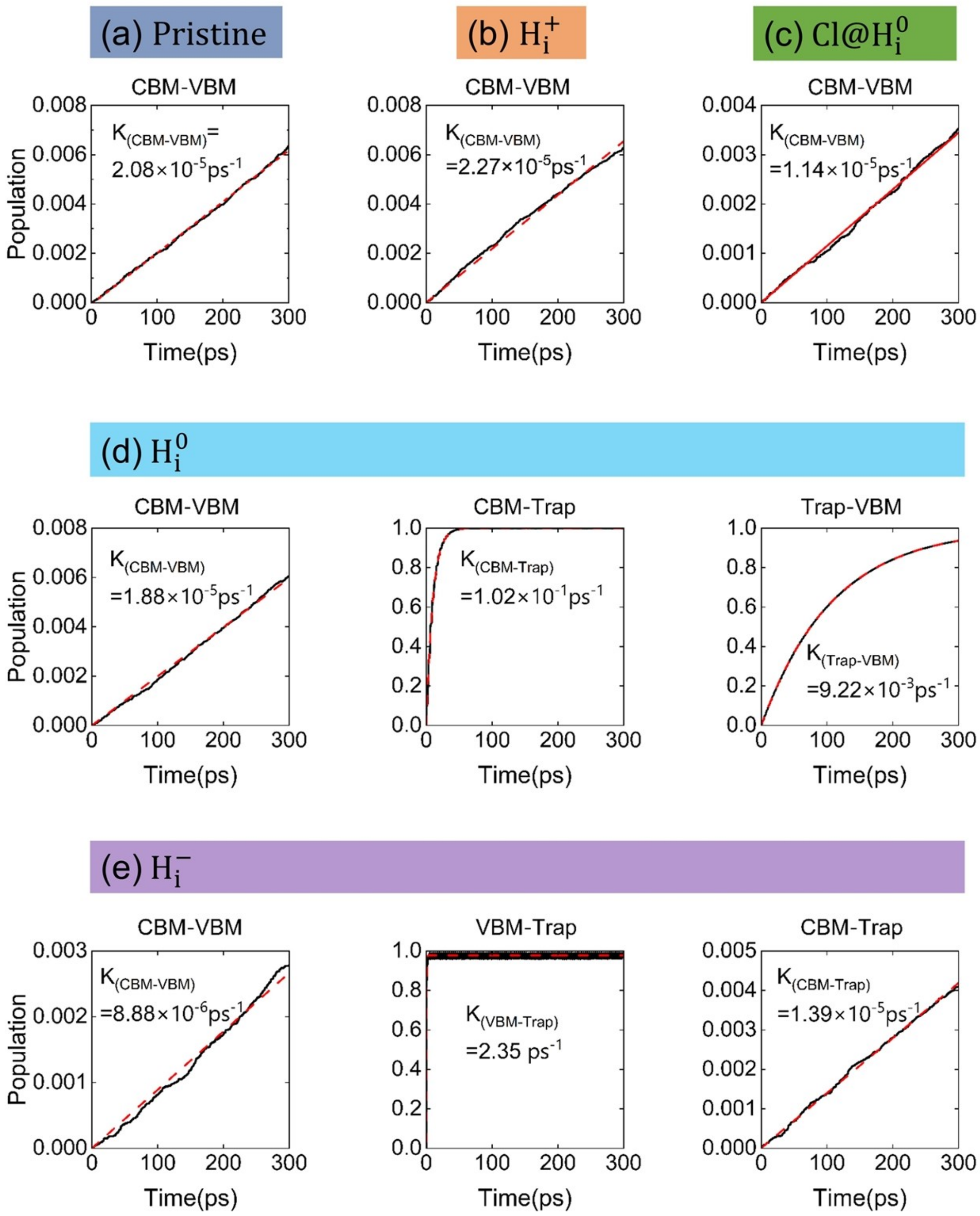

Figure S4. Time evolution of electronic state populations obtained from nonadiabatic molecular dynamics simulations for (a) pristine $FAPbI_3$, (b) H_i^+, (c) Cl@H_i^0, (d) H_i^0, (e) H_i^- system. The population transfer between CBM, VBM, and trap states is shown, and the corresponding recombination or carrier capture rate constants k are obtained from linear fitting.

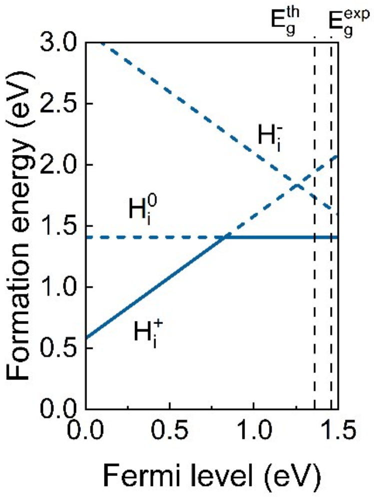

Figure S5. Formation energy of H_i as a function of Fermi level.
